# Establishing cytogenetic biodosimetry laboratory in Saudi Arabia and producing preliminary calibration curve of dicentric chromosomes as biomarker for medical dose estimation in response to radiation emergencies

**DOI:** 10.1007/s13205-014-0217-x

**Published:** 2014-04-18

**Authors:** Khaled Al-Hadyan, Sara Elewisy, Belal Moftah, Mohamed Shoukri, Awad Alzahrany, Ghazi Alsbeih

**Affiliations:** 1 Radiation Biology Section, King Faisal Specialist Hospital & Research Centre (KFSHRC), Riyadh, Kingdom of Saudi Arabia; 2 Biomedical Physics Department, King Faisal Specialist Hospital & Research Centre (KFSHRC), Riyadh, Kingdom of Saudi Arabia; 3 Biotechnology Centre, King Faisal Specialist Hospital & Research Centre (KFSHRC), Riyadh, Kingdom of Saudi Arabia; 4 Atomic Energy Research Institute, King Abdulaziz City for Science and Technology, Riyadh, Kingdom of Saudi Arabia; 5 Radiation Biology Section, Biomedical Physics Department, King Faisal Specialist Hospital & Research Centre (KFSHRC), P.O. Box 3354, MBC-03, Riyadh, 11211 Kingdom of Saudi Arabia

**Keywords:** Biodosimetry, Radiation overexposure, Cytogenetics, Dicentric chromosomes, Dose–response calibration curve

## Abstract

In cases of public or occupational radiation overexposure and eventual radiological accidents, it is important to provide dose assessment, medical triage, diagnoses and treatment to victims. Cytogenetic bio-dosimetry based on scoring of dicentric chromosomal aberrations assay (DCA) is the “gold standard” biotechnology technique for estimating medically relevant radiation doses. Under the auspices of the National Science, Technology and Innovation Plan in Saudi Arabia, we have set up a biodosimetry laboratory and produced a national standard dose–response calibration curve for DCA, pre-required to estimate the doses received. For this, the basic cytogenetic DCA technique needed to be established. Peripheral blood lymphocytes were collected from four healthy volunteers and irradiated with radiation doses between 0 and 5 Gy of 320 keV X-rays. Then, lymphocytes were PHA stimulated, Colcemid division arrested and stained cytogenetic slides were prepared. The Metafer4 system (MetaSystem) was used for automatic and manually assisted metaphase finding and scoring of dicentric chromosomes. Results were fit to the linear-quadratic dose–effect model according to the IAEA EPR-Biodosimetry-2011 report. The resulting manually assisted dose–response calibration curve (*Y* = 0.0017 + 0.026 × *D* + 0.081 × *D*^2^) was in the range of those described in other populations. Although the automated scoring over-and-under estimates DCA at low (<1 Gy) and high (>2 Gy) doses, respectively, it showed potential for use in triage mode to segregate between victims with potential risk to develop acute radiotoxicity syndromes. In conclusion, we have successfully established the first biodosimetry laboratory in the region and have produced a preliminary national dose–response calibration curve. The laboratory can now contribute to the national preparedness plan in response to eventual radiation emergencies in addition to providing information for decision makers and public health officials who assess the magnitude of public, medical, occupational and accidental radiation exposures.

## Introduction

The beneficial applications of radiation in medicine, agriculture, energy, industry and research greatly improve the quality of our daily life. Therefore, the Kingdom of Saudi Arabia has launched the atom for peace initiative to profit from the tremendous capabilities of nuclear sciences while ensuring international confidence in its peaceful applications (Aljohani 2008; Fineren 2013). However, the increase in various radiological applications will collaterally be associated with increased probability of instances in which one or more individuals will accidentally be overexposed (Turai et al. 2002; González 2007). In any scenario, radiation protection directives dictate the establishment of emergency response capability for rapid medical diagnosis and management of overexposed individuals (Turai et al. 2004; WHO 2003; Beinke et al. 2013).

Cytogenetic abnormalities are one of the most striking and consistent effects of ionizing radiation on living organisms. When the energy associated with ionizing radiation is transferred to molecules in cells, the DNA that embeds the genetic materials is damaged in proportion to the type and amount of energy that is absorbed. In human lymphocytes, this leads to the appearance of structurally abnormal chromosomes when cells attempt to divide following radiation exposure. Between the different types of chromosomal aberrations induced, dicentric chromosomes appear to be more specific to radiation exposure with a background level practically equal to zero. Hence, the number of dicentrics is quantified and compared to a calibration dose–response curve, established in vitro to derive an estimate of possible dose received. This strategy is valid because lymphocytes express the damage regardless of whether they are irradiated in vivo or in vitro. Therefore, the cytogenetic dicentric chromosomal assay (DCA) became the internationally recommended method for biological dosimetry by International Organization for Standardization (ISO 2004) and International Atomic Energy Agency (IAEA 2001). It uses the genetic effect of ionizing radiation on human body and relies on the frequency of dicentric chromosomal aberrations found in metaphases from cultured human peripheral blood lymphocyte.

In cases of individual radiation overexposure, it is important to provide suitable dose assessment, medical triage, diagnoses and treatment to victims. The accepted generic approach for effective medical management of a suspected acute radiation overexposure incident necessitates recording dynamic medical data, performing appropriate radiation bioassays for dose estimation, and measuring radioactivity to provide diagnostic information to the treating physician and a dose assessment for personnel radiation protection records (Alexander et al. 2007; Blakely et al. 2009). These are achieved by observing and recording prodromal symptoms and signs, obtaining complete blood counts with white blood cell differentials, measuring physical dose from personal dosimeters if available, and sampling blood for cytogenetic chromosome aberration using the “gold standard” DCA, which is the corner stone in radiation bioassays. Furthermore, in the event of a radiological mass-casualty incident, national and also international resources need to be enhanced to provide suitable dose assessment and medical triage and diagnoses (Sullivan et al. 2013). Therefore, many nations have established deployable reference expert, cytogenetic biodosimetry laboratories as part of the medical responder community and national radiation protection program (Blakely et al. 2009; Miller et al. 2007; Voisin et al. 2002; Beinke et al. 2013).

In line with the radiation protection directives, we have initiated a program to establish a national biodosimetry laboratory that has been funded by “The Long-Term Comprehensive National Plan for Science, Technology and Innovation”, currently known as the “National Science Technology and Innovation Program (NSTIP)” administered by “King Abdulaziz City for Science and Technology (KACST)”. The primary objective is to establish a national standard dose–response calibration curve for DCA, pre-required to estimate doses received in cases of accidental radiation overexposure. To achieve this goal, the cytogenetic DCA technique needed first to be set up. In this report, we describe the establishment of the technique and evaluate the yield of dicentric chromosomal aberrations in peripheral lymphocytes irradiated in vitro in four Saudi individuals, in our effort to ultimately produce the in-house dose–response calibration curve, which is the benchmark of the biodosimetry laboratory for the formation of national radiation emergency response capability in Saudi Arabia.

## Materials and methods

### Equipment

The automated metaphase finder “Metafer4 system” mounted on the AxioImager.Z2 microscope (manufacturer: MetaSystems/Carl Zeiss, Germany) forms the corner stone of equipment needed for this study. Basic configuration and software were purchased and used for auto-capture of metaphases using the AutoCapt module while dicentric aberration scoring was performed with the image analysis system module DCScore (MetaSystems). The basic equipment did not include option for fluorescence, thus experiment was restricted to classic Giemsa-stained cytogenetic preparation.

## Volunteers, blood samples and irradiation

Four healthy Saudi volunteers, aged 23, 24, 34 and 40 years old, were recruited for this study. The Basic Research and the Ethics Committees of the institutional review board have approved the project. Following signing an informed consent, 20 ml peripheral blood samples were taken by routine venipuncture in heparinized tubes (Vacuette, Greiner Bio-One GmbH, Germany) and were transferred to 10 × 25 ml cell culture flasks (2 ml each) kept at 37 °C to receive single X-rays radiation dose of either 0, 0.10, 0.25, 0.50, 0.75, 1, 2, 3, 4, or 5 Gy. Irradiation was performed using X-RAD 320 (Precision X-ray, CT, USA) biological irradiator at a maximum energy of 320 keV filtered with 2 mm Al, and a dose rate of 1.33 Gy/min. In addition to ionizing chamber (PTW, Freiburg, Germany), the absorbed dose was also measured using a GAFCHROMIC film (EBT2 model) as described previously (Aldelaijan et al. 2013).

## Lymphocytes culture

Following 2-h of incubation at 37 °C, pre-warmed 18 ml of complete RPMI-1,640 (with l-glutamine; Sigma-Aldrich, USA) media complemented with 1 % of 100× Penicillin–Streptomycin antibiotic solution (100 IU and 100 µg per ml, respectively, Sigma-Aldrich), 15 % Fetal Bovine Serum (Hyclone, ThermoScientific, USA) and 400 µl phytohemagglutinin (PHA, Remel Europe Ltd, ThermoScientific, USA) to each flask to stimulate lymphocytes division. Culture flasks were incubated for 48 h at 37 °C, 5 % CO_2_ atmosphere. Colcemid was added (final concentration of 0.10 µg/ml; Irvine Scientific, CA, USA) for the final 4 h to arrest cell division at metaphases.

## Hypotonic and cell fixation procedures

Cultures were transferred to 50 ml tubes and centrifuged at 1,100 RPM (200 g) for 8 min. Cells were resuspended and 10 ml of hypotonic solution (0.075 M KCl) was added gently and incubated for 12 min at room temperature (RT). Soft fixation was carried out by adding 2 ml of fresh fixative (3:1 methanol/acetic acid), and let to stand for 10 min at RT. Cells were centrifuged, resuspended and 10 ml fresh fixative was added and let to stand for 10 min at RT for two cycles, after which they were stored at −20 °C for at least 30 min prior to slide preparation.

## Metaphases slide preparation and Giemsa staining

Cells were centrifuged (200*g*, 8 min) and resuspended in appropriate amount of fixative so as to have slightly cloudy appearance that ensures appropriate cell concentration. Metaphase spreads were prepared by dropping 40 µl with a pipette on a pre-cleaned and moistened slide and were dried on a slide-warmer set at 40 °C. Staining was performed with 10 % Giemsa solution in phosphate buffer (pH 6.8) for 10 min (4 ml Giemsa + 36 ml PBS pH 6.8 in a coplin jar), then rinsed in distilled water and air dried before being mounted with Eukitt (Fluka, Sigma-Aldrich) medium and coverslipped.

## Analysis of slides

We have used the automated Metafer4 system (MetaSystems, Altlussheim, Germany) for autocapture of metaphases including finding, image acquisition, storing and relocation. Dicentric aberration scoring was performed after exporting the stored images into an image analysis system (DCScore, MetaSystems). This software recognizes and scores uniquely dicentric chromosomes using a trainable classifier. Other types of aberrations such as acentric fragments and ring chromosomes were manually tracked where needed. Only complete metaphases with 46 centromeres were included in the analysis, which was performed by two staff members. Metaphases were classified as having 0, 1, 2, 3, 4, 5, or 6 dicentric according to the number of dicentric chromosomes found. Furthermore, occasionally observed tricentric aberrations were scored as two dicentrics. Two methods were used, manually assisted (also known as semi-manual) and fully automated scoring as described by the manufacturer of Metafer4 system. The manually assisted scoring is carried out by reviewing images of the fully automated files to ascertain validity of scores. The *x*- and *y*-stage coordinates were used to permit relocation and re-examination as needed. Four to five slides per data point were scanned and results were pooled to obtain sufficient number of metaphase (≥100 for the highest radiation dose) for data analysis.

## Statistical analysis

Detailed information about cytogenetic biodosimetry data analysis is available in IAEA Technical Report 405 (IAEA 2001). The analysis of the yield of dicentrics in metaphase spreads included an evaluation of the distribution of dicentrics using the Papworth test as described previously (IAEA 2011). The yield of dicentric chromosomes per radiation dose was calculated by dividing the total number of dicentrics found by the entire number of metaphases counted. Then, the dose–effect relationship was determined for dicentric chromosomes. Adequate curve fitting requires a sufficient number of degrees of freedom; therefore, ten doses (including zero) were included in the dose range according to ISO guidelines (ISO 2004). Data were fitted using the linear-quadratic dose–response curve (*Y* = *C* + *αD* + *βD*^2^) by the method of maximum likelihood (Frome and DuFrain 1986; Merkle 1983) using the statistical software package “R”. The coefficients of the fitted curves, the intercept *C* and the linear *α* and the quadratic *β* components were derived for each respective individual and for all collectively pooled for each radiation dose. The goodness-of-fit was evaluated by a scaled deviance method (McCullagh and Nelder 1989). The 95 % confidence interval on the fitted curve was computed assuming Poisson distribution (which stipulates that the values of standard *U* test of the goodness-of-fit described by Papworth and adopted by Savage (Papworth and Savage 1975) are included between ±1.96, thus if the magnitude of the *U* value is out of that range, the dispersion of dicentrics is significant at the 5 % level). For inter-individuals differences, a Chi square homogeneity test was performed for dicentrics frequencies. The free CABAS (Chromosome Aberration Analysis Software, Version 2.0, http://www.ujk.edu.pl/ibiol/cabas/), developed at the Swietokrzyska Academy, Kielce, Poland (Deperas et al. 2007), was used for the estimation of dose in hypothetical scenario of radiation over-exposure. This software is specifically designed for biological dosimetry based on the analysis of chromosome aberrations (dicentrics and rings) scored from Giemsa-stained slides and uses the maximum likelihood method to fit calibration data to the linear-quadratic equation.

## Results

After establishing the cytogenetic dicentric assay (DCA), and training staff in the laboratory, four healthy volunteers were recruited to determine the dose–response calibration curve for dicentric induction by X-rays exposure, as a prerequisite for retrospective radiation dose assessment. The volunteers were healthy at the time of blood donation, with no known history of diseases or drug use besides active smoking of one volunteer. The median age was 29 years/old which is a representative average age of active radiological workers who might be at risk for accidental exposure. Peripheral blood lymphocytes were irradiated with 320 keV X-rays, and dicentric yields were determined in first-division metaphase spreads obtained from 48-h blood cultures. The Metafer4 system was used to auto-capture metaphases, then scores and analyzes chromosomal aberrations particularly dicentric (frequently ascertained by the presence of acentric fragment). Although the main results presented here are those obtained by the manually assisted (or semi-manual) scoring, results of fully automated scoring were also carried out and discussed for their potential use in triage mode in cases of mass-causality accident.

**Fig. 1:**
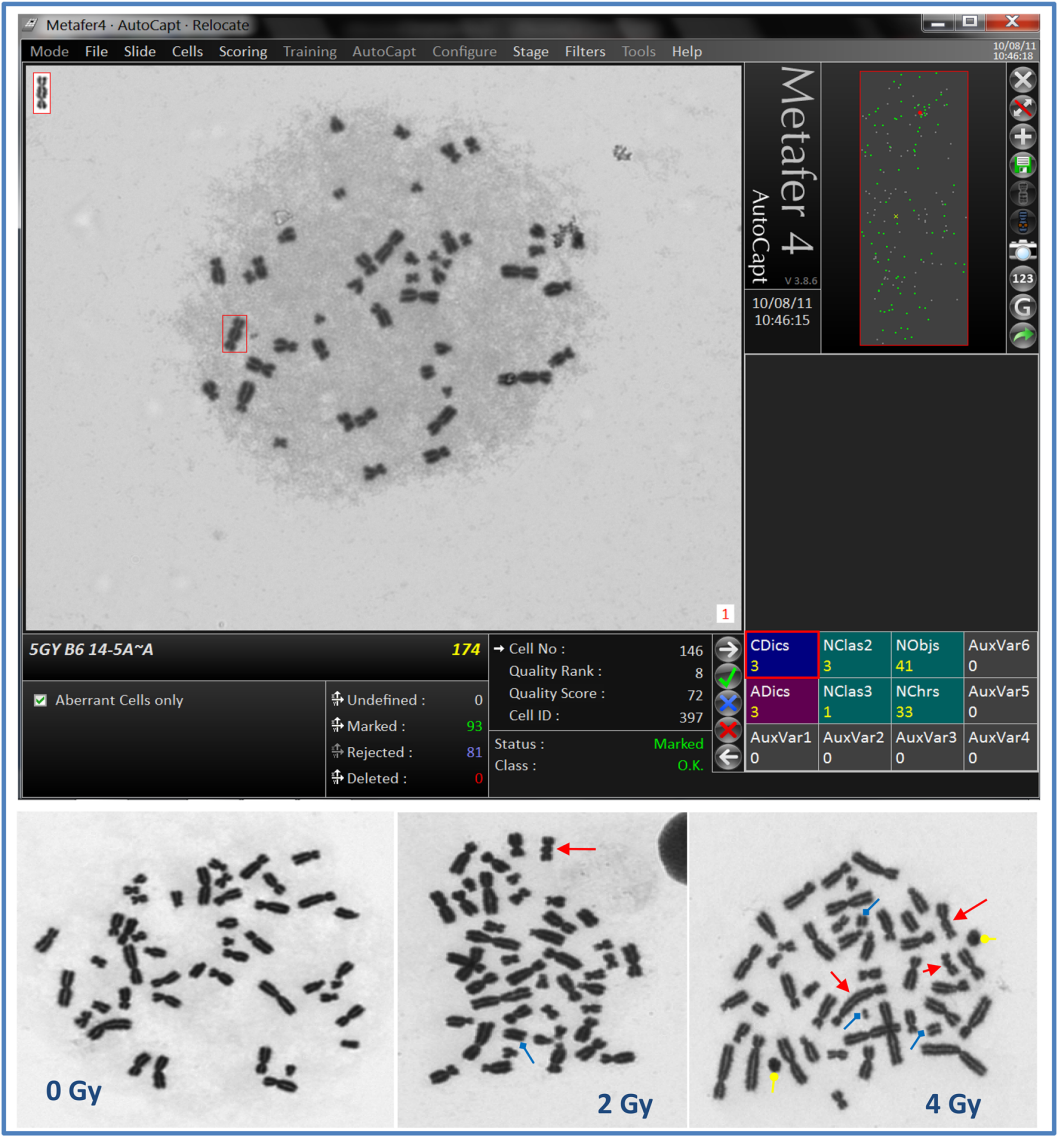
Representative example of metaphase with dicentric chromosome captured by the Metafer4 system (*upper panel*) and normal metaphase in control (0 Gy) and dicentric (*arrow*), acentric fragments (*squared arrow head*) and rings (*rounded arrow head*) in irradiated lymphocytes (*lower panel*)

**Fig. 2:**
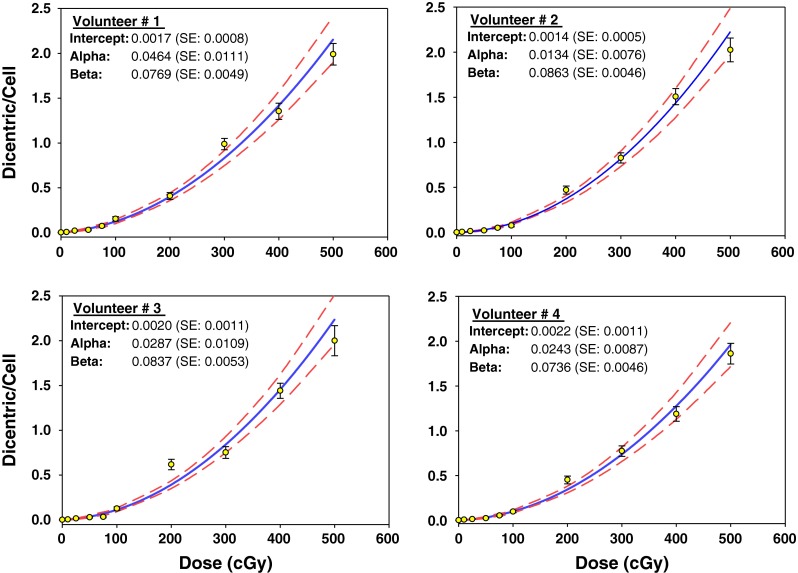
Linear-quadratic dose–response curves (*solid lines*) for dicentric chromosomal aberrations induced by 320 keV X-rays in lymphocytes derived from four Saudi volunteers. Data points represent the yield of dicentric per metaphase scored using manually assisted mode. *Broken lines* are the 95 % confidence limits calculated assuming Poisson distribution. *Error bars* represent the standard errors

Illustrative example of Metafer4 screen snapshot along with normal and aberrations containing metaphases is shown in Fig. 1. Results of biodosimetric dicentric scoring are given in Table 1, which shows the numbers of metaphase spreads analyzed, dicentrics observed, and the average number of dicentrics per metaphase in each individual. As expected, there was a steep decrease in the number of metaphases recorded with increasing dose that was offset by relative increase in the number of dicentric observed. Thus, while about a thousand of metaphases were possible to record in each sample at the lower radiation dose, in compliance with IAEA recommendations at least 100 dicentrics were scored at the highest 5 Gy dose in the 4–5 slides prepared. The results indicate that the yields of dicentric increase rapidly with dose in a manner that is comparable between the four individuals at each radiation dose. The homogeneity test on the residual mean squared error of the fitted curves (*X*^2^ = 0.000017, 3 degrees of freedom, *P* = 0.99) indicated no significant differences between them. The similarity can further be appreciated in the resulting dose–effect relationship of individuals’ dicentric yields shown in Fig. 2. The individual dose–response curve fitted to the linear-quadratic model indicates that, for example, a dose of 2.5 Gy would yield 0.5985, 0.5745, 0.5972, and 0.5231 dicentric per metaphase in each of the four individuals, i.e., an average of 0.5733 (SD = 0.035). Thus, it is justifiable to pool these data together to obtain a general dose–response calibration curve representative of a priori healthy individuals in Saudi Arabia that is in compliance with IAEA recommendations on the number of metaphases and/or dicentrics scored and as generally practiced in this field [see for example (Beinke et al. 2010)].

**Table 1:** Yield and intercellular distribution of dicentric chromosomal aberrations after in vitro X-rays irradiation of blood samples derived from four Saudi individuals

Dose (Gy)	Individual	N. metaphases	N. dicentrics	*D*0*	*D*1*	*D*2*	*D*3*	*D*4*	*D*5*	*Y*	DI	*U* value
0	1	2,229	4	2,225	4	0	0	0	0	0.002	1.00	−0.05
	2	4,572	5	4,567	5	0	0	0	0	0.001	1.00	−0.05
	3	1,476	3	1,473	3	0	0	0	0	0.002	1.00	−0.05
	4	1,591	3	1,588	3	0	0	0	0	0.002	1.00	−0.04
0.1	1	1,220	8	1,212	8	0	0	0	0	0.007	0.99	−0.15
	2	1,346	8	1,338	8	0	0	0	0	0.006	0.99	−0.14
	3	755	5	750	5	0	0	0	0	0.007	0.99	−0.12
	4	1,087	8	1,079	8	0	0	0	0	0.007	0.99	−0.16
0.25	1	609	13	596	13	0	0	0	0	0.021	0.98	−0.36
	2	926	13	914	13	0	0	0	0	0.014	0.99	−0.29
	3	618	10	609	10	0	0	0	0	0.016	0.99	−0.27
	4	1,014	14	1,001	14	0	0	0	0	0.014	0.99	−0.30
0.5	1	535	16	519	16	0	0	0	0	0.030	0.97	−0.47
	2	759	17	743	17	0	0	0	0	0.022	0.98	−0.42
	3	432	13	419	13	0	0	0	0	0.030	0.97	−0.43
	4	703	17	688	17	0	0	0	0	0.024	0.98	−0.44
0.75	1	395	29	370	27	1	0	0	0	0.073	1.00	−0.02
	2	656	33	625	29	2	0	0	0	0.050	1.07	1.33
	3	438	15	423	15	0	0	0	0	0.034	0.97	−0.49
	4	651	35	616	35	0	0	0	0	0.054	0.95	−0.96
1	1	234	36	204	32	2	0	0	0	0.154	0.96	−0.39
	2	538	41	500	35	3	0	0	0	0.076	1.07	1.20
	3	345	44	304	38	3	0	0	0	0.128	1.01	0.16
	4	537	53	484	53	0	0	0	0	0.099	0.90	−1.60
2	1	267	109	178	70	18	1	0	0	0.408	0.98	−0.22
	2	240	113	148	74	16	1	1	0	0.471	0.98	−0.27
	3	215	133	132	46	37	3	1	0	0.619	1.18	1.87
	4	230	104	144	71	12	3	0	0	0.452	0.96	−0.47
3	1	173	171	45	91	24	8	2	0	0.988	0.70	−2.79
	2	220	182	76	85	30	11	1	0	0.827	0.87	−1.35
	3	186	140	92	58	28	6	2	0	0.753	1.08	0.79
	4	203	157	92	71	34	6	0	0	0.773	0.89	−1.07
4	1	144	195	35	49	40	15	4	1	1.354	0.87	−1.08
	2	142	214	6	41	37	16	9	3	1.507	0.76	−2.03
	3	156	225	25	53	43	19	6	1	1.442	0.78	−1.97
	4	149	177	38	51	40	10	4	0	1.188	0.83	−1.45
5	1	104	207	6	43	26	20	8	4	1.990	0.76	−1.76
	2	78	158	1	26	20	15	8	3	2.026	0.66	−2.10
	3	69	138	11	17	16	13	10	2	2.000	0.97	−0.17
	4	87	162	2	25	27	16	5	3	1.862	0.62	−2.51

*N. metaphases* number of cells in metaphase assessed, *N. dicentrics* total number of dicentrics found in the metaphases assessed, *Y* yield of dicentrics, i.e., the number of dicentrics per metaphase (cell), *DI* dispersion index, *U value* a *U* value between −1.96 and +1.96 indicates a poisson distribution

* Number of metaphases with 0, 1, 2, 3, 4, 5 dicentrics, respectively

**Fig. 3:**
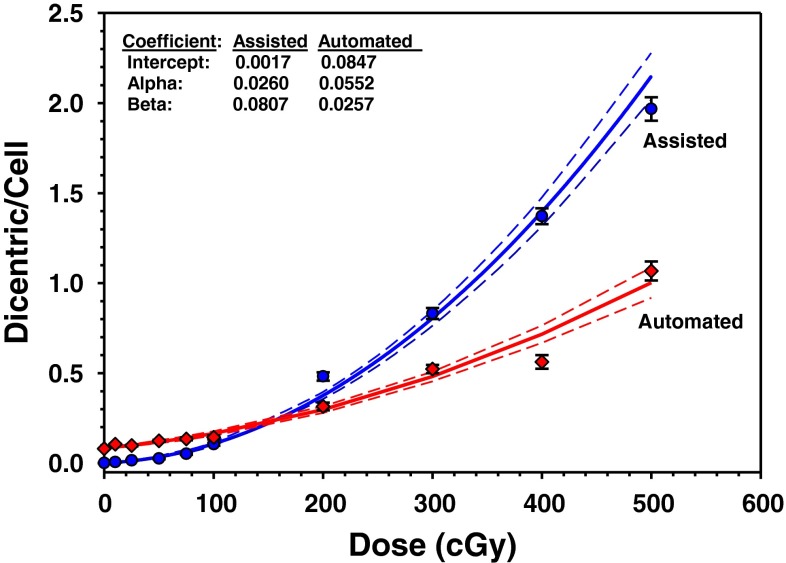
Linear-quadratic dose–response calibration curves (*solid lines*) for dicentric chromosomal aberrations induced by 320 keV X-rays in lymphocytes derived from four Saudi volunteers. Data points represent the yield of dicentric per metaphase scored using either manually assisted (*circles*) or automated (*diamonds*) mode. *Broken lines* are the 95 % confidence limits calculated assuming Poisson distribution. *Error bars* represent the standard errors

Pooled results of dicentric chromosomal aberration of the four individuals are given in Table 2. In total, there were 3,028 dicentric chromosomes found in 26,329 analyzed metaphase spreads (average yield of 0.12 dicentric per metaphases). The background level of dicentric chromosomes determined by the analysis of 9,868 metaphase spreads from unirradiated blood samples was about two dicentric per 1,000 metaphases. After exposure to radiation doses ranging from 0.10 to 5.0 Gy, the number of dicentric increased with some metaphases displaying 2, 3, 4 and even 5 dicentric chromosomes (see Fig. 1). This was associated with steep decrease in the number of metaphases that could be scored with increasing dose (more than 10-fold decrease, from 4,408 for 0.10 Gy to reach 338 for 5 Gy). On the other hand, the yield of dicentric had steeply increased from 0.007 to 1.967 (Table 2). The preliminary dose–response calibration curve for dicentric chromosomal aberrations generated from the pooled results is shown in Fig. 3. The curve has a classic linear-quadratic shape with all data points within or very close to the 95 % confidence interval limits calculated assuming Poisson distribution. The goodness-of-fit for the curve for dicentric induction (scaled deviance, i.e., deviance/degree of freedom, *P* = 0.001) indicates an excellent fit (McCullagh and Nelder 1989). The fitted coefficients using the statistical software “R” were *Y* = 0.0017 (±0.0004) + 0.0260 (±0.0046) × *D* + 0.0807 (±0.0024) × *D*^2^).

**Table 2:** Yield and intercellular distribution of dicentric chromosomal aberrations induced in peripheral blood lymphocytes by X-rays exposure

Dose (Gy)	N. metaphases	N. dicentrics	*D*0*	*D*1*	*D*2*	*D*3*	*D*4*	*D*5*	*Y*	DI	*U* value
0	9,868	15	9,853	15	0	0	0	0	0.002	0.99	−0.10
0.1	4,408	29	4,379	29	0	0	0	0	0.007	0.99	−0.30
0.25	3,167	50	3,120	50	0	0	0	0	0.016	0.98	−0.62
0.5	2,429	63	2,369	63	0	0	0	0	0.026	0.97	−0.89
0.75	2,140	112	2,034	106	3	0	0	0	0.052	1.00	0.06
1	1,654	174	1,492	158	8	0	0	0	0.105	0.98	−0.35
2	952	459	602	261	83	8	2	0	0.482	1.03	0.86
3	782	650	305	305	116	31	5	0	0.831	0.88	−2.29
4	591	811	104	194	160	60	23	5	1.372	0.82	−2.97
5	338	665	20	111	89	64	31	12	1.967	0.73	−3.42

Results of four healthy Saudi individuals

*N. metaphases* number of cells in metaphase assessed, *N. dicentrics* total number of dicentrics found in the metaphases assessed, *Y* yield of dicentrics, i.e., the number of dicentrics per metaphase (cell), *DI* dispersion index, *Uvalue* a *U* value between −1.96 and +1.96 indicates a poisson distribution

* Number of metaphases with 0, 1, 2, 3, 4, 5 dicentrics, respectively

The pooled results of the Metafer4 fully automated scoring are presented in Table 3. Overall, there were clearly higher number of dicentrics scored (4,898) in a total of 29,667 metaphases counted (average yield of 0.17 dicentric per metaphases). The resulting dose–response calibration curve was compared to manually assisted scoring (Fig. 3). A homogeneity test based on Chi square showed that the two curves are significantly different from each other (*X*^2^ = 55.63, 2 degrees of freedom, *P* < 0.05). The fitted coefficients for automated scoring were *Y* = 0.0847 (±0.0023) + 0.0552 (±0.0072) × *D* + 0.0257 (±0.0023) × *D*^2^). There were an over-estimation of dicentric yield at low doses below 1 Gy and under-estimation at high doses above 2 Gy.

**Table 3:** Metafer automated scoring of dicentric chromosomal aberrations induced in vitro in human lymphocytes by X-rays exposure

Dose (Gy)	N. metaphases	N. dicentrics	*D*0*	*D*1*	*D*2*	*D*3*	*D*4*	*D*5*	*Y*	DI	*U* value
0	10,956	883	10,150	746	47	11	0	2	0.081	1.15	10.81
0.1	4,190	443	3,777	387	22	4	0	0	0.106	1.05	2.20
0.25	2,815	276	2,560	234	21	0	0	0	0.098	1.05	2.05
0.5	2,354	293	2,091	236	24	3	0	0	0.124	1.10	3.48
0.75	2,370	320	2,070	281	18	1	0	0	0.135	1.00	−0.12
1	2,292	331	1,990	275	25	2	0	0	0.144	1.04	1.47
2	2,076	655	1,553	418	83	18	3	1	0.315	1.19	6.09
3	1,415	740	987	459	178	61	20	4	0.523	1.97	25.89
4	640	360	371	199	53	13	4	0	0.562	1.08	1.50
5	559	597	186	208	116	39	10	0	1.068	0.92	−1.42

Pooled data of blood samples derived from four healthy individuals

*N. metaphases* number of cells in metaphase assessed, *N. dicentrics* total number of dicentrics found in the metaphases assessed, *Y* yield of dicentrics, i.e., the number of dicentrics per metaphase (cell), *DI* dispersion index, *U value* a *U* value between −1.96 and +1.96 indicates a poisson distribution

* Number of metaphases with 0, 1, 2, 3, 4, 5 dicentrics, respectively

## Discussions

The main aim of this study was to establish the dicentric chromosome assay (DCA) in our radiation biology laboratory and to produce a dose–response calibration curve for Saudi individuals. As most probable radiological accidents are expected to occur due to the external exposure to γ-rays sources and X-rays producing radiation equipment, the first aim was to determine the national dose–response calibration curve for dicentric induction by low-LET ionizing radiation as prerequisites to build cytogenetic biodosimetry laboratory in Saudi Arabia and to provide first responder capabilities. To date, the DCA remains the gold standard for retrospective dosimetry after whole-body or partial-body exposure in acute and recent radiation accidents. Further development will include other types of radiations and also wider range of cellular and molecular biomarkers of radiation exposure currently in active research (Rothkamm et al. 2013).

In this study, we report our successful experience in biodosimetry and make the first preliminary national dose–response calibration curve for dicentric chromosomal aberrations induced by 320 keV X-rays available for the scientific community. The study involved peripheral blood samples from four healthy volunteers aged between 23 and 40 years old. Blood samples, irradiation, cytogenetic preparation and analysis followed essentially protocols described previously (IAEA 2011). The automated Metafer4 system (MetaSystems, Germany) was successfully used to capture metaphases and score dicentrics in two modes, fully automated and manually assisted whereby validity of scoring was ascertained by scorer. The latter mode was preferred throughout this study as dicentrics were confirmed visually and had been frequently associated with acentric fragments. Although the automated mode appeared to require further improvement and better adjustment of the Metafer4 classifier, still it has applications in triage mode in cases of mass-causality incidents where it can quickly provide diagnostic screening tool to discriminate between victims with high- and low-radiation exposure risk.

An important observation in this study, often overlooked in the literature, is to highlight that the four volunteers included in this study showed comparable yield of dicentrics induction by X-rays in lymphocytes (Table 1), confirmed by the lack of statistically significant differences (homogeneity test, *P* = 0.99). This has resulted in essentially comparable linear-quadratic dose–response curves (Fig. 2). Although testing more volunteers is required to confirm this conclusion, it suggests low variability between individuals implying that results are generalizable to the related population.

The resulting preliminary dose–response calibration curves pooled from the four volunteers showed a classical linear-quadratic shape (Fig. 3). The yield of dicentrics steadily increased with dose from 0.10 to 5 Gy. The 0.10 Gy showed a yield that is distinguishable from the background level and, therefore, it could be considered the lower limit of the assay (Table 2). Further enhancement, however, can be brought about by improving statistical significance of the background level of dicentrics as it was relatively higher than that reported frequently in the literature of one dicentric per 1,000 metaphases (IAEA 2011; Lloyd et al. 1980; Beinke et al. 2010) and further optimizations are in progress in our laboratory.

**Fig. 4:**
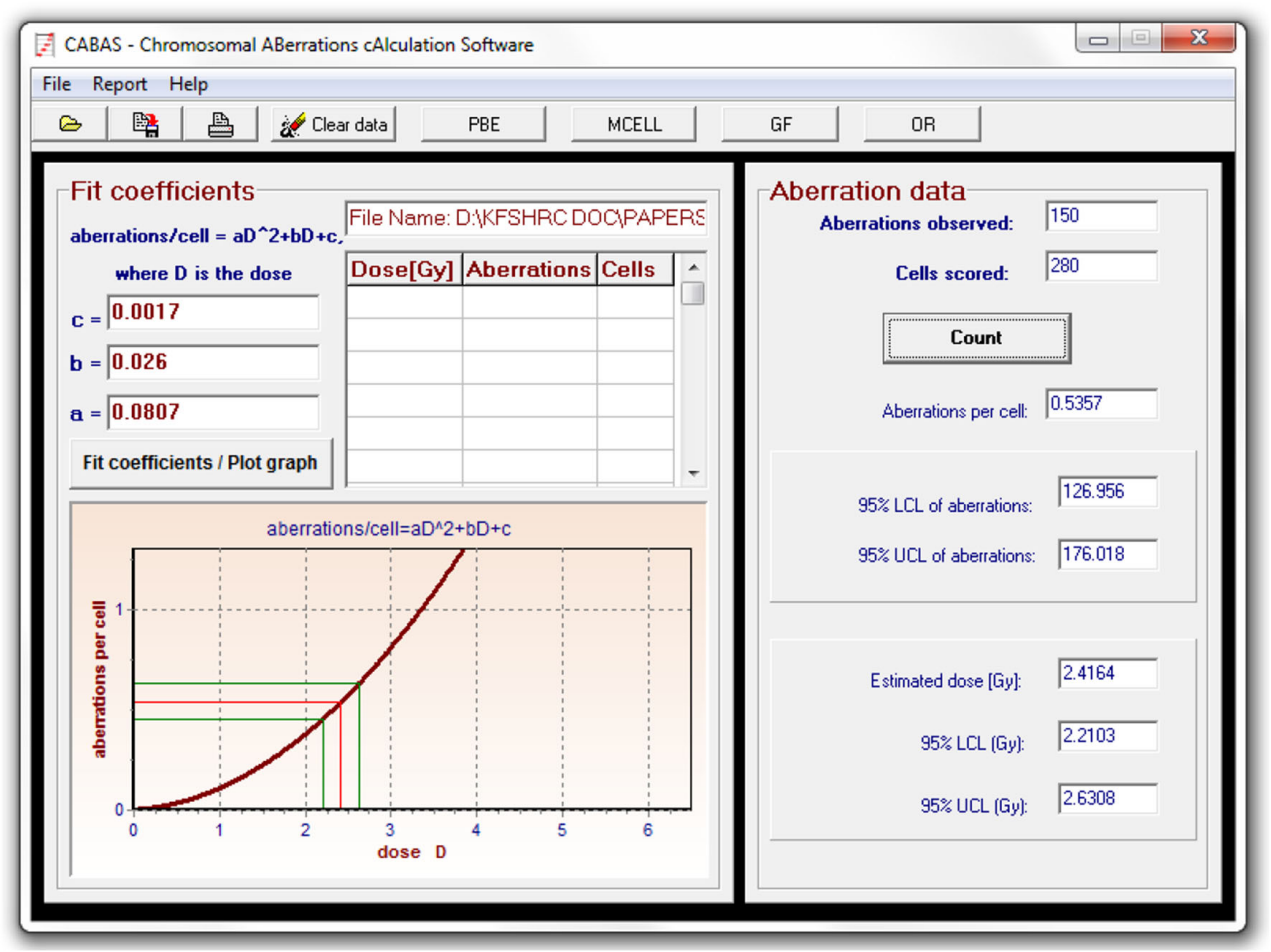
Illustrative practical example of estimating radiation dose received, with its 95 % confidence interval, in a scenario of total body irradiation using CABAS software. Utilizing the coefficients of the national calibration curve, a hypothetical accidental over-exposure that yields, for example, 150 dicentric per 280 metaphases, would be caused by an absorbed dose of 2.416 Gy with a lower and upper 95 % confidence limits of 2.21 and 2.63 Gy, respectively

Comparing the coefficients of the dose–response relationship for dicentric induction (*Y* = 0.0017 + 0.0260 × *D* + 0.0806 × *D*^2^) with those from similar published studies (Schmid et al. 1984; Barquinero et al. 1997; IAEA 2011; Beinke et al. 2010), good general agreement can be observed. However, some inter-laboratory variations exist, which could result from the energy of irradiation, the dose rate, methodical or technical differences, scoring criteria, and the experience of the scorers. Nonetheless, the preliminary dose–response calibration curve in Saudi Arabia is in the range of those published in other population (Wilkins et al. 2008; Beinke et al. 2010; Martins et al. 2013; Lee 2011; Lee et al. 2012). For example, while the yield of dicentric induced by a dose of 2 Gy, ranged between 0.21 and 0.48 (mean = 0.33, SD = 0.099) in other populations, it was 0.48 in Saudi Arabia. This shows an upper-range dose–response calibration curve suggesting that people in Saudi Arabia are in agreement with cytogenetic radiosensitivity when compared to other population. Thus, this calibration curve can now be used to estimate radiation dose received in cases of accidental radiation over-exposure. For example, using CABAS software (Deperas et al. 2007), a radiation dose received in a hypothetical accidental over-exposure that yields, for example, 150 dicentric per 280 metaphases (this is an average estimation from our experiments with the four volunteers), i.e., an yield of 0.5357 dicentric per metaphase, would be caused by an absorbed dose of 2.416 Gy with a lower and upper 95 % confidence limits of 2.21 and 2.63 Gy, respectively (Fig. 4).

Although the two calibrations curves obtained by the manually assisted (or semi-manual) and the automated dicentrics and metaphases scoring modes showed differences (Fig. 3), results presented here further validate the use of automated scoring for triage purposes (Romm et al. 2013). The criteria for triage purposes stipulate performing dose assessments on the analysis of various 50-metaphase spreads (Lloyd et al. 2000). The accuracy is considered to be sufficient under a preliminary triage in a mass-casualty event. In such an emergency circumstances, the output of biodosimetry triage needed by a physician falls into four exposure categories related to dose interval: (A) 0–1.0 Gy, (B) 1.0–3.5 Gy, (C) 3.5–5.0 Gy and (D) above 5.0 Gy. The automated mode can give valuable diagnostic information to segregate between these various risk groups. Of particular importance, the automated mode can give results that are very close to those obtained by the manually assisted mode in the critical exposure range between 1 and 3 Gy which can be used to screen between victims with low risk not requiring urgent medical attention from high-risk exposure requiring immediate therapeutic intervention with probability to develop acute hematopoietic radiation syndrome.

## Conclusion

We have successfully established the first biological dosimetry laboratory in Saudi Arabia and in the region and have produced a preliminary national dose–response calibration curve for dicentric chromosomal aberrations induced by 320 keV X-rays. The calibration curve was in range of those described in other population. The laboratory can now estimate radiation doses received in eventual accidental radiation exposures as part of a national preparedness plan in response to radiation emergencies in addition to providing information for decision makers and public health officials who assess the magnitude of public, medical and occupational irradiation.
